# Supplementary material for: Multivariable Mendelian Randomization With Weak Instruments: A Comparison of Bayesian and Frequentist Methods
Source: Stat Med. 2026 Oct 18;45(23-24):e70788. doi: 10.1002/sim.70788 (PMC13678027; doi:10.1002/sim.70788)
Supplement: Supplementary file 1 [file SIM-45-0-s001.pdf]

Supplementary material to: Multivariable Mendelian randomization with weak instruments: a comparison of Bayesian and frequentist methods

Andrew J. Grant[1], Ashish Patel[2], and Stephen Burgess[2,3]

[1]Sydney School of Public Health, University of Sydney, Sydney, Australia
[2]MRC Biostatistics Unit, University of Cambridge, Cambridge, UK
[3]Cardiovascular Epidemiology Unit, University of Cambridge, Cambridge, UK

S1 Simulation setup with three exposures

Correlated genetic effecs

To simulate scenarios with three exposures and correlated genetic effects, we generated data from (1) and (2) with $K = 3$, $\theta_1 = 0.5$, $\theta_2 = 0$, $\theta_3 = 0.2$, $J = 60$, $\text{var}(\varepsilon_Y) = \text{var}(\varepsilon_{Xk}) = \text{var}(\varepsilon_U) = 1$, and the genetic variant-exposure associations distributed as

$$\begin{pmatrix} \beta_{Xj1} \\ \beta_{Xj2} \\ \beta_{Xj3} \end{pmatrix} \sim N\left(\begin{pmatrix} 0.1 \\ 0.1 \\ 0.1 \end{pmatrix}, 0.04^2 \begin{pmatrix} 1 & \rho & \rho \\ \rho & 1 & \rho \\ \rho & \rho & 1 \end{pmatrix} \right). \tag{S1}$$

As in the primary simulation setting, ρ was varied between 0 and 0.8.

Measurement error

To simulate scenarios with measurement error on three exposures, we again generated data from (1) and (2) with $K = 3$, $\theta_1 = 0.5$, $\theta_2 = 0$, $\theta_3 = 0.2$, $J = 60$, and $\text{var}(\varepsilon_Y) = \text{var}(\varepsilon_{Xk}) = \text{var}(\varepsilon_U) = 1$. The genetic variant-exposure associations were distributed as in (S1) with $\rho = 0.2$. The genetic association estimates were taken from a regression of the instruments on $X_1^* = X_1 + \zeta_1$, $X_2^* = X_2 + \zeta_2$, and $X_3^* = X_3 + \zeta_3$, with ζ_1, ζ_2, and ζ_3 independently drawn from the $N(0, \nu)$ distribution. As in the primary simulation setting, ν was varied between 0 and 4.

Mediation

To simulate scenarios with mediation, we generated the exposures according to

$$X_1 = \sum_{j=1}^{J} \beta_{Xj1} G_j + U + \varepsilon_{X1}$$
$$X_2 = \alpha X_1 + \sqrt{1-\alpha^2} \left(\sum_{j=1}^{J} \beta_{Xj2} G_j + U + \varepsilon_{X2} \right)$$
$$X_3 = \sum_{j=1}^{J} \beta_{Xj3} G_j + U + \varepsilon_{X3},$$

with $J = 60$ and $\text{var}(\varepsilon_Y) = \text{var}(\varepsilon_{Xk}) = \text{var}(\varepsilon_U) = 1$. The genetic variant-exposure associations were distributed as in (S1) with $\rho = 0.2$. The outcome was generated according to (2) with $\theta_1 = \theta_2 = 0.5$, $\theta_3 = 0.2$, and $\text{var}(\varepsilon_Y) = 1$. As in the primary simulation setting, α was varied between 0 and 0.8.

Supplementary figures

S1 Simulation results for the estimation of θ_1 in the supplementary scenarios with increasing correlated genetic effects. 4
S2 Simulation results for the estimation of θ_2 in the supplementary scenarios with increasing correlated genetic effects. 5
S3 Simulation results for the estimation of θ_1 in the supplementary scenarios with increasing measurement error. 6
S4 Simulation results for the estimation of θ_2 in the supplementary scenarios with increasing measurement error. 7
S5 Simulation results for the estimation of θ_1 in the supplementary scenarios with increasing level of mediation. 8
S6 Simulation results for the estimation of θ_2 in the supplementary scenarios with increasing level of mediation. 9
S7 Simulation results for the primary simulation scenario with increasing correlated genetic effects and reduced number of instruments. 10
S8 Simulation results for the primary simulation scenario with increasing measurement error on exposures and reduced number of instruments. 11
S9 Simulation results for the primary simulation scenario with increasing level of mediation and reduced number of instruments. 12
S10 Simulation results for the primary simulation scenario with increasing correlated genetic effects and increased number of exposures. 13
S11 Simulation results for the primary simulation scenario with increasing measurement error on exposures and increased number of exposures. 14
S12 Simulation results for the primary simulation scenario with increasing level of mediation and increased number of exposures. 15

Supplementary tables

S1 Simulation results for the estimation of θ_1 in the primary scenario with increasing correlated genetic effects 16
S2 Simulation results for the estimation of θ_2 in the primary scenario with increasing correlated genetic effects 17
S3 Simulation results for the estimation of θ_1 in the primary scenario with increasing measurement error on exposures 18
S4 Simulation results for the estimation of θ_2 in the primary scenario with increasing measurement error on exposures 19
S5 Simulation results for the estimation of θ_1 in the primary scenario with increasing level of mediation 20
S6 Simulation results for the estimation of θ_2 in the primary scenario with increasing level of mediation 21

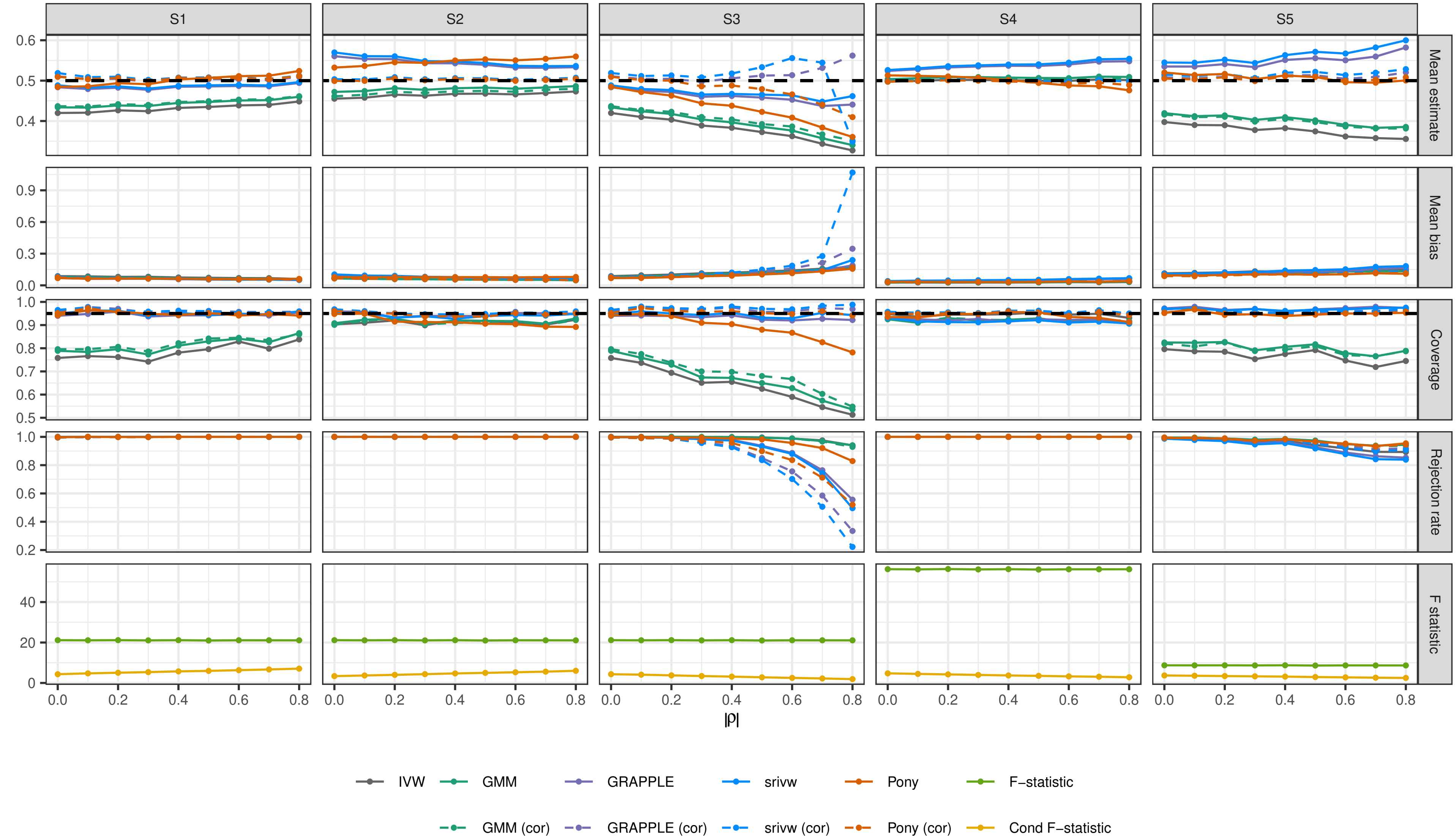

Figure S1. Simulation results for the estimation of θ_1 in the supplementary scenarios with increasing correlated genetic effects.

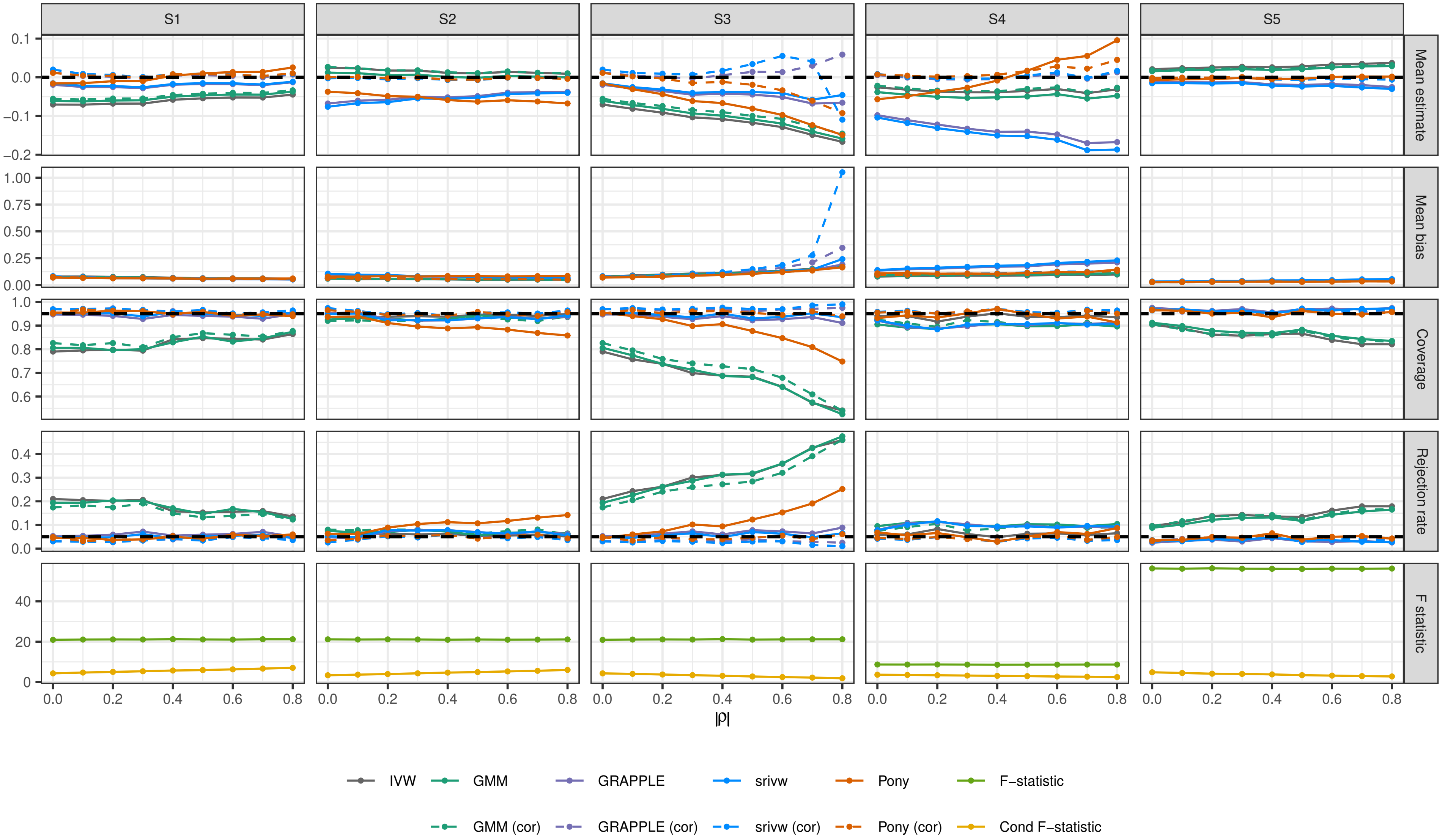

Figure S2. Simulation results for the estimation of θ_2 in the supplementary scenarios with increasing correlated genetic effects.

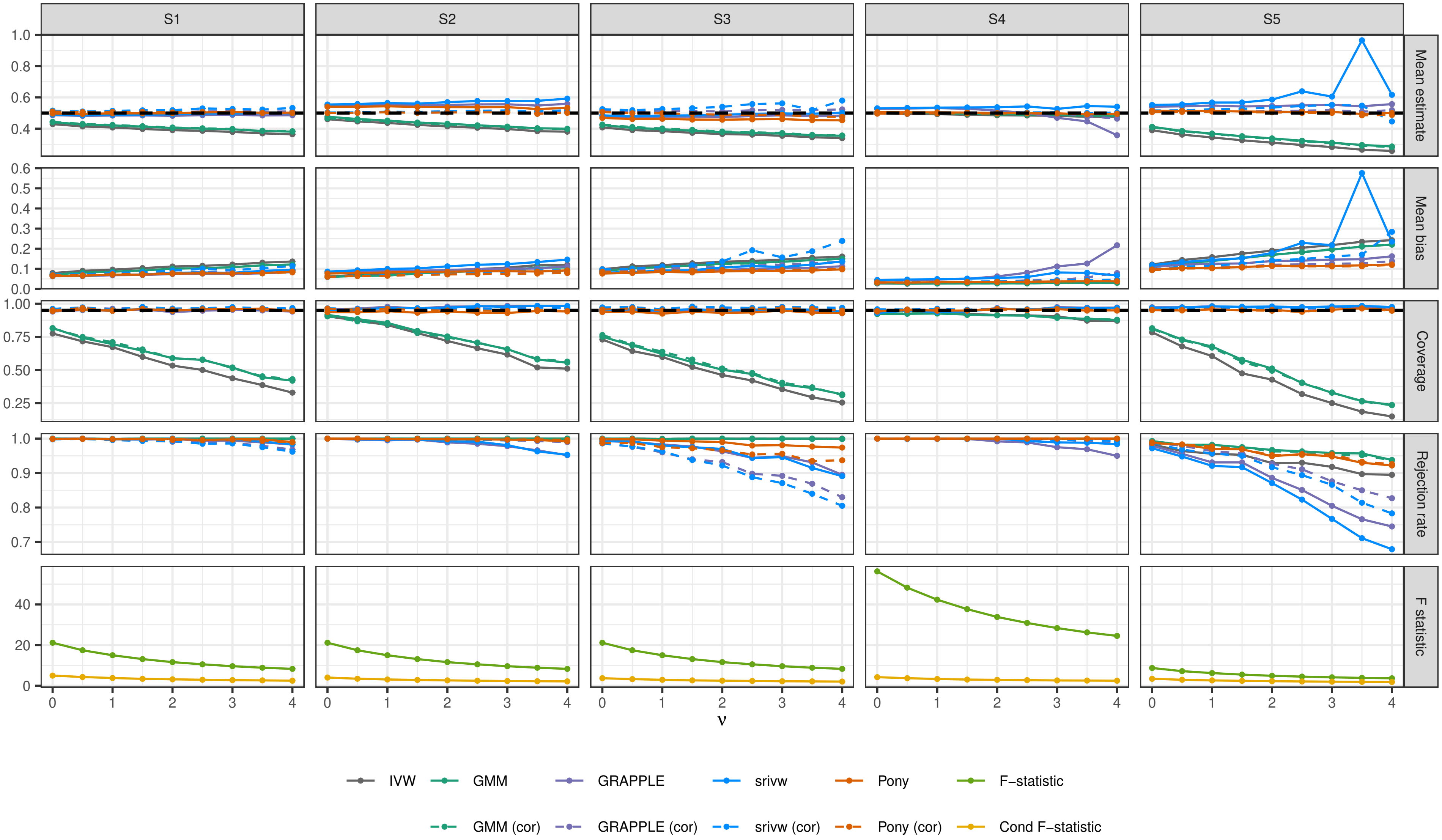

Figure S3. Simulation results for the estimation of θ_1 in the supplementary scenarios with increasing measurement error.

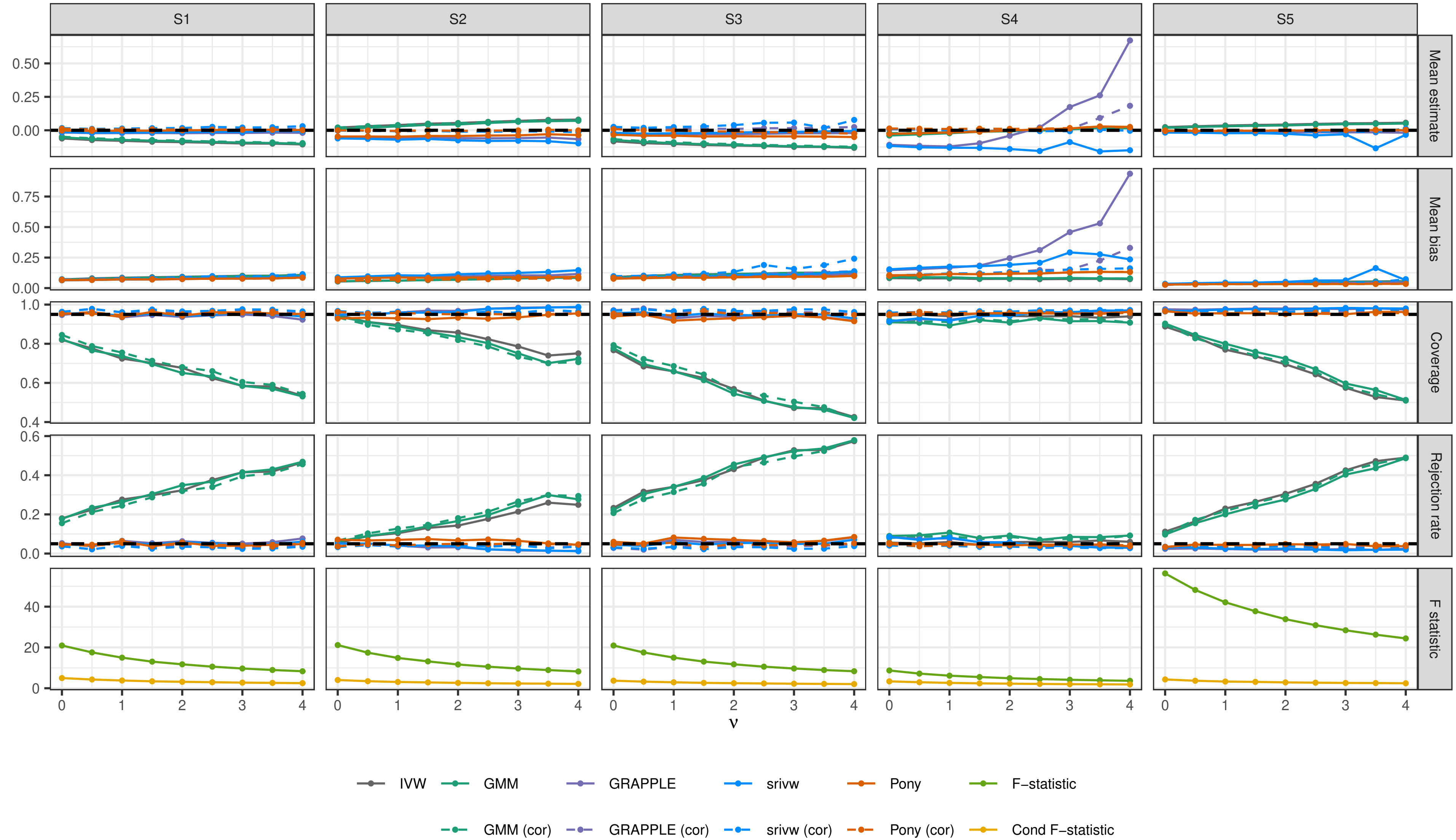

Figure S4. Simulation results for the estimation of θ_2 in the supplementary scenarios with increasing measurement error.

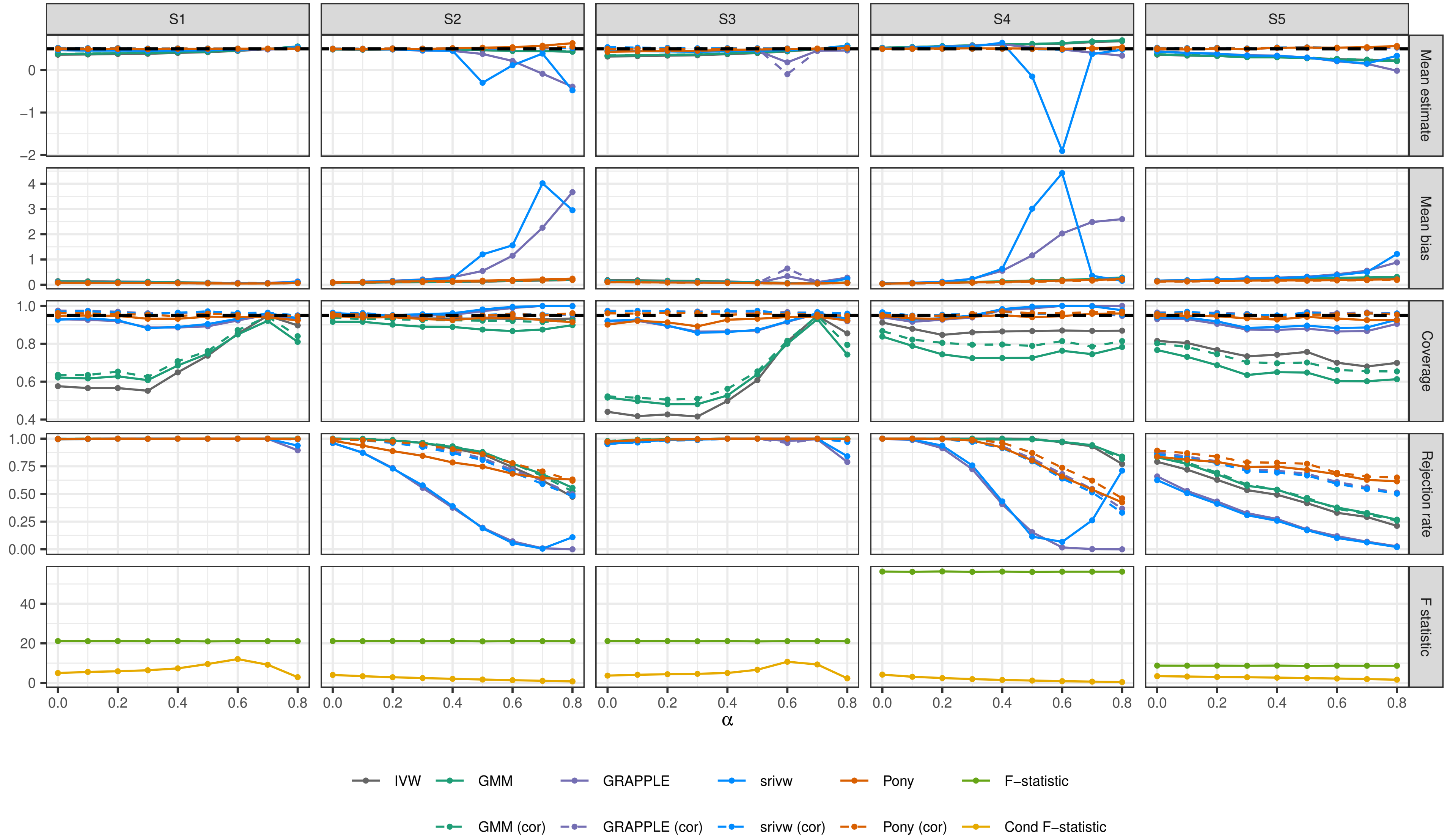

Figure S5. Simulation results for the estimation of θ_1 in the supplementary scenarios with increasing level of mediation.

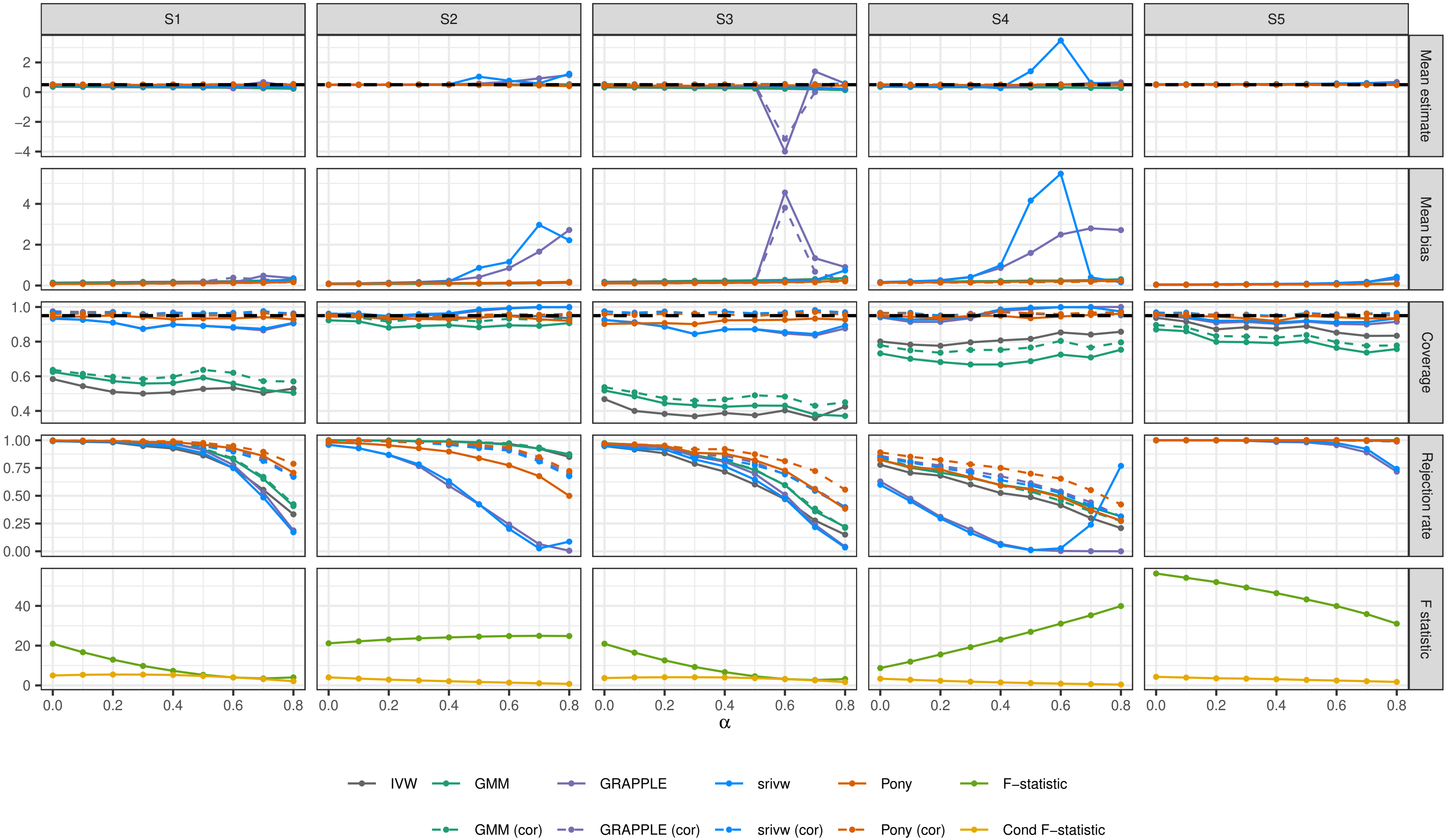

Figure S6. Simulation results for the estimation of θ_2 in the supplementary scenarios with increasing level of mediation.

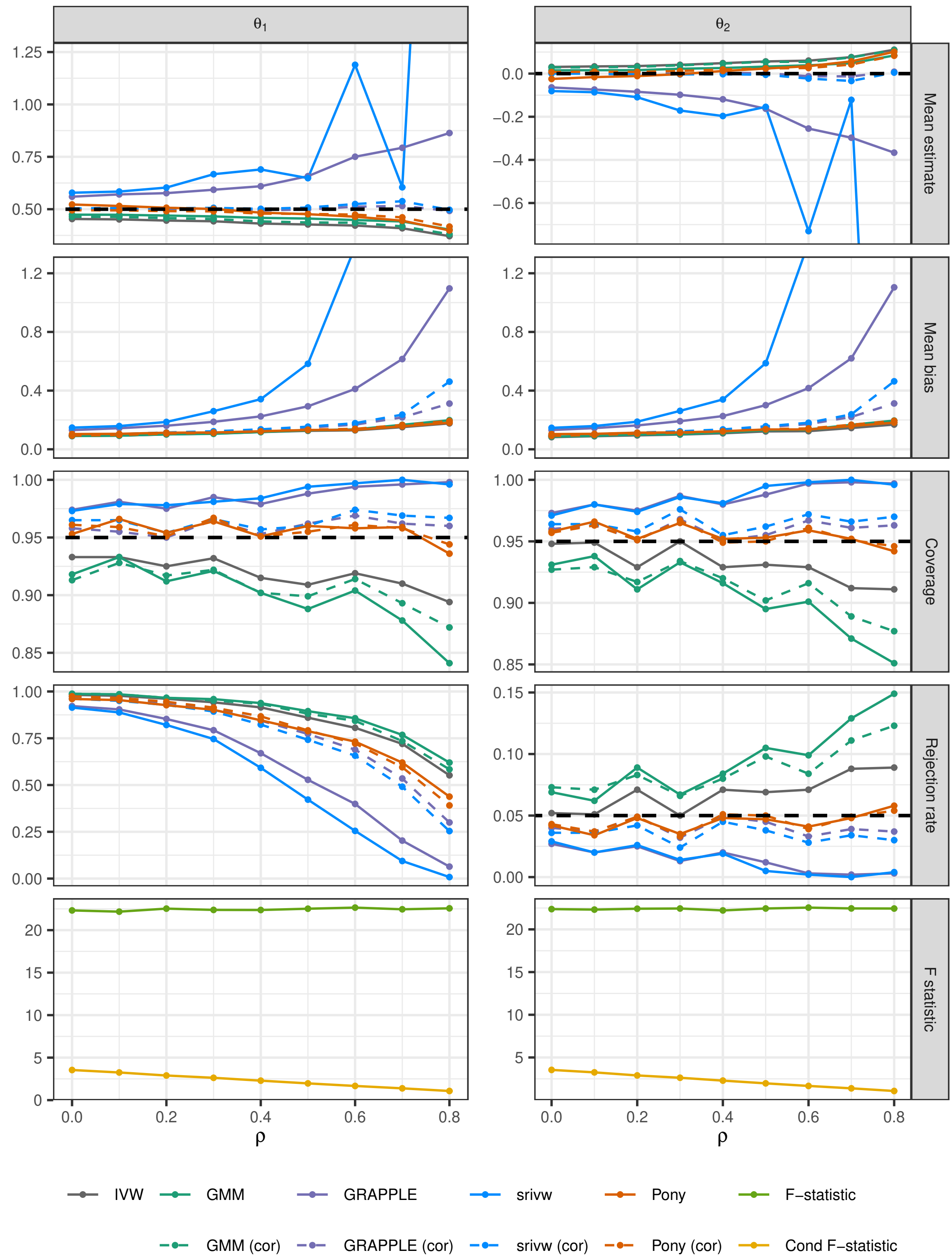

Figure S7. Simulation results for the primary simulation scenario with increasing correlated genetic effects and reduced ($J = 30$) number of instruments. Note that some values for the srivw method are beyond the displayed range.

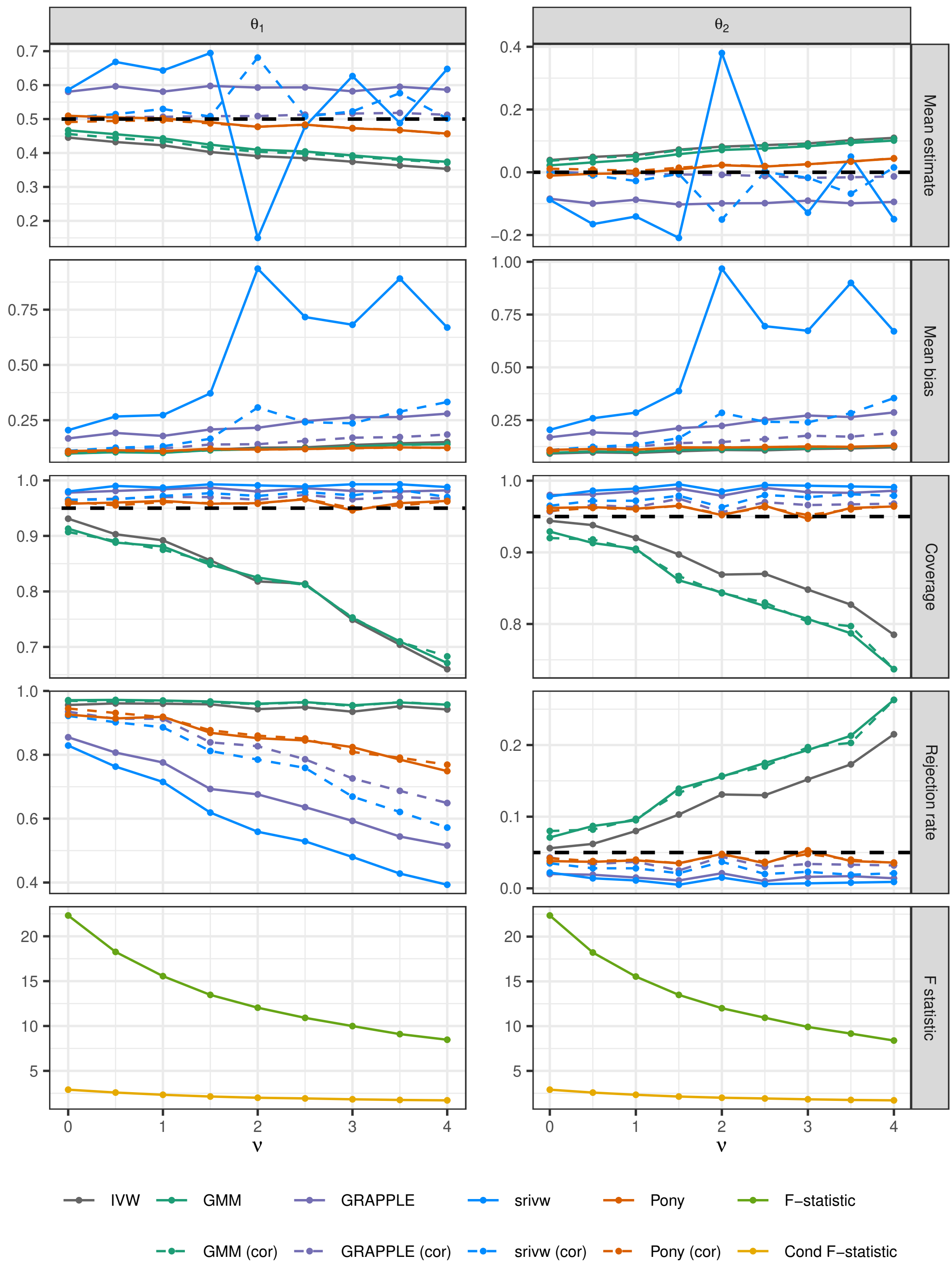

Figure S8. Simulation results for the primary simulation scenario with increasing measurement error on exposures and reduced ($J = 30$) number of instruments.

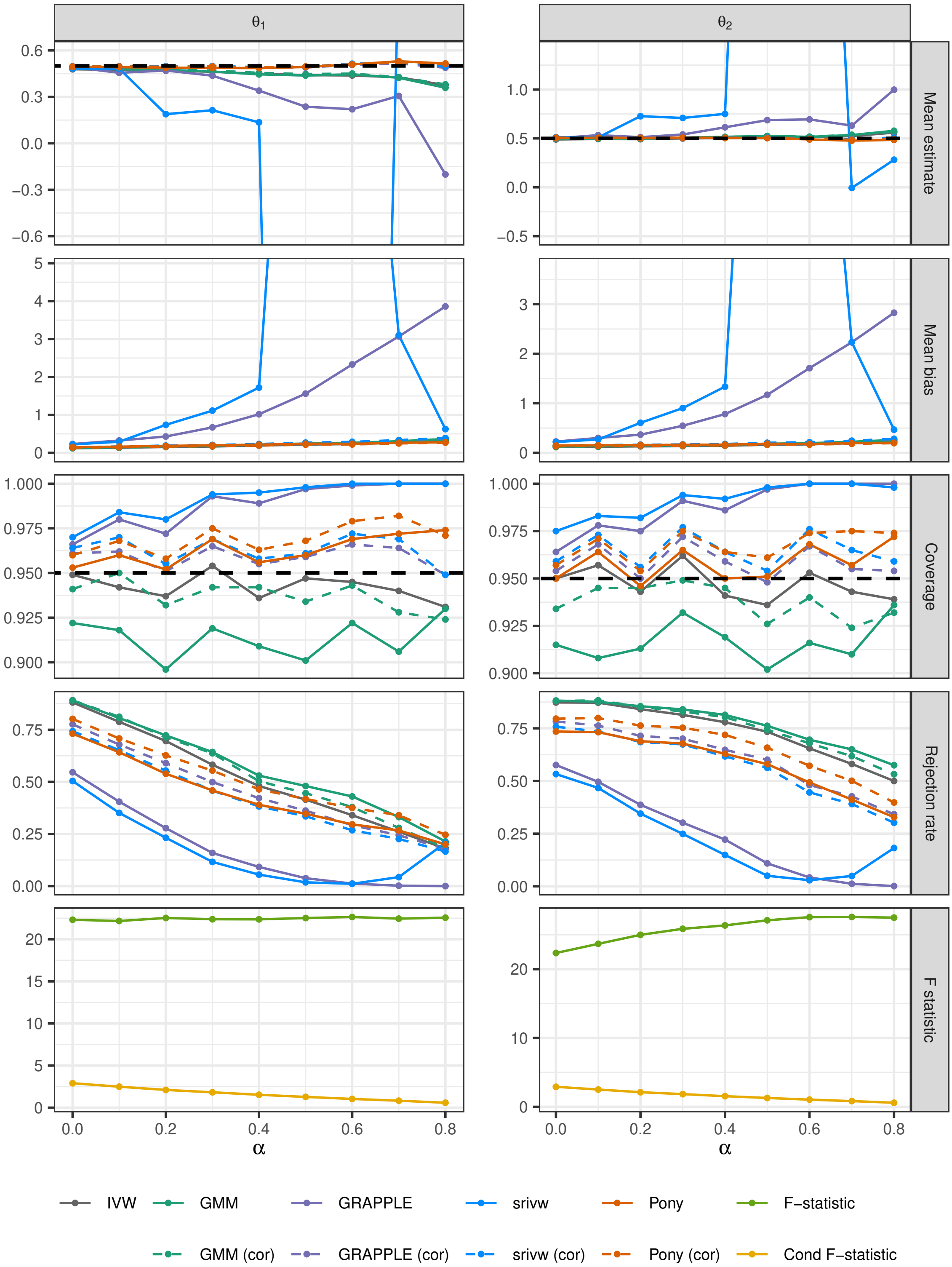

Figure S9. Simulation results for the primary simulation scenario with increasing level of mediation and reduced ($J = 30$) number of instruments. Note that some values for the srivw method are beyond the displayed range.

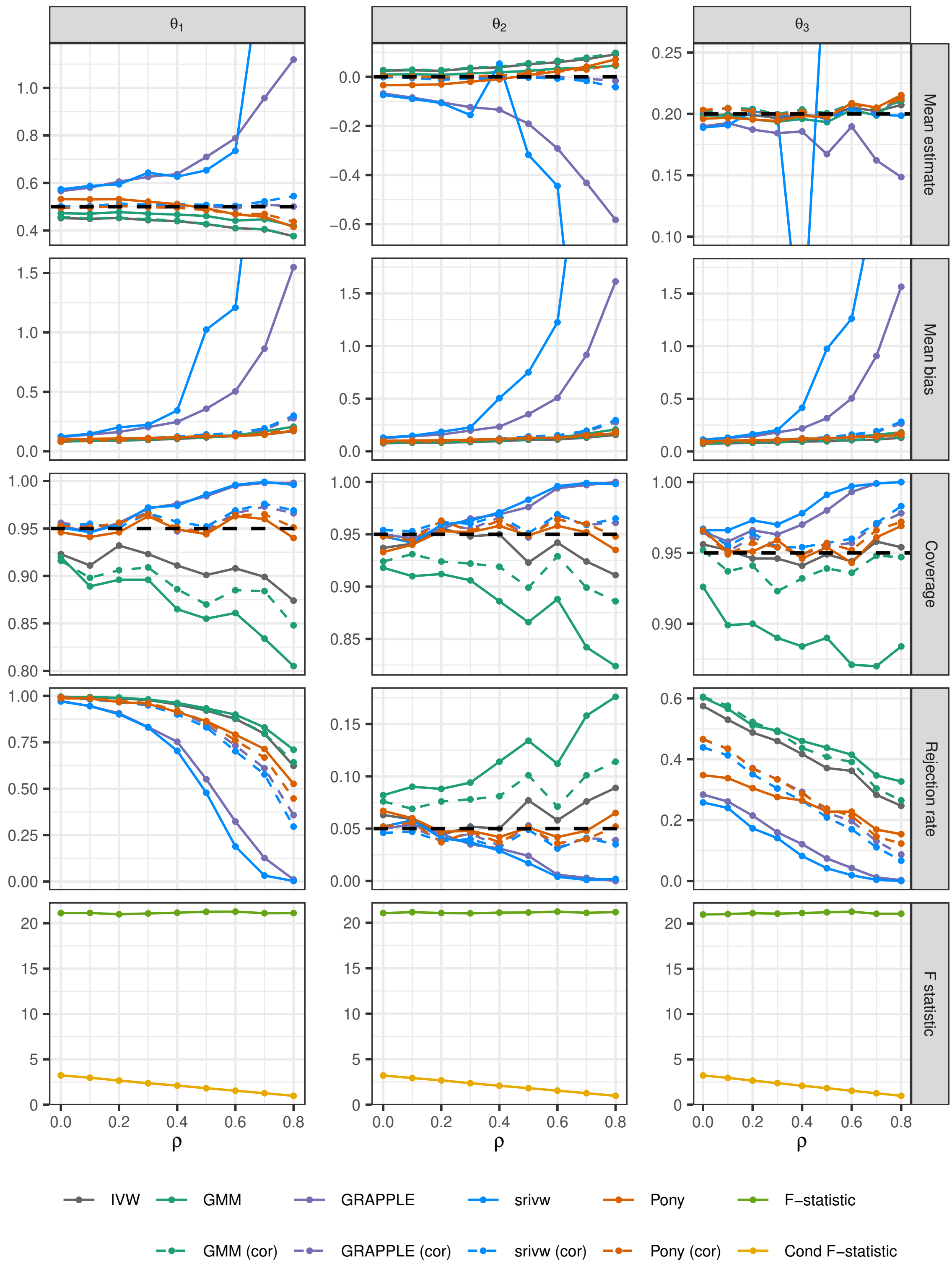

Figure S10. Simulation results for the primary simulation scenario with increasing correlated genetic effects and increased ($K = 3$) number of exposures. Note that some values for the srivw method are beyond the displayed range.

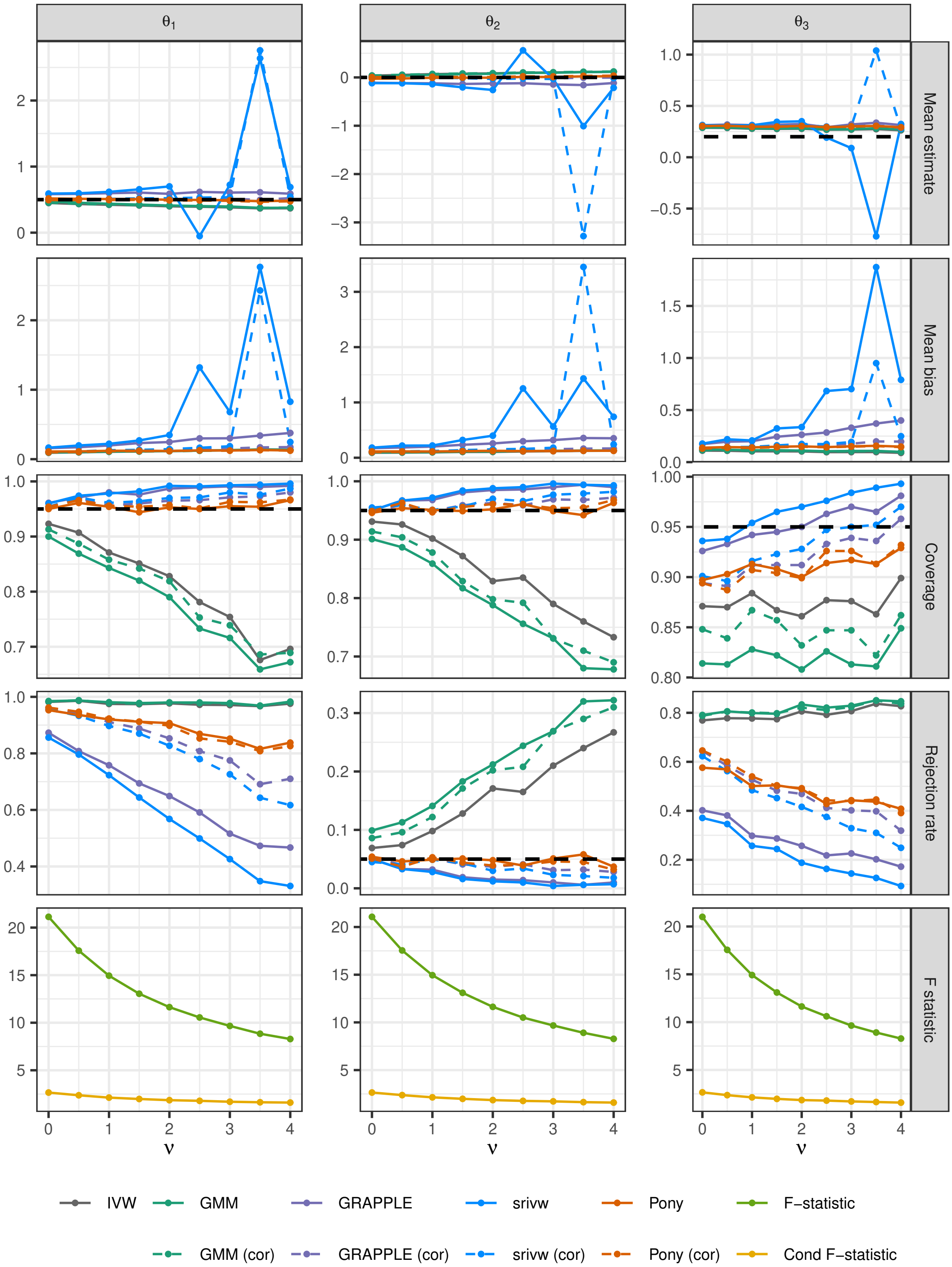

Figure S11. Simulation results for the primary simulation scenario with increasing measurement error on exposures and increased ($K = 3$) number of exposures.

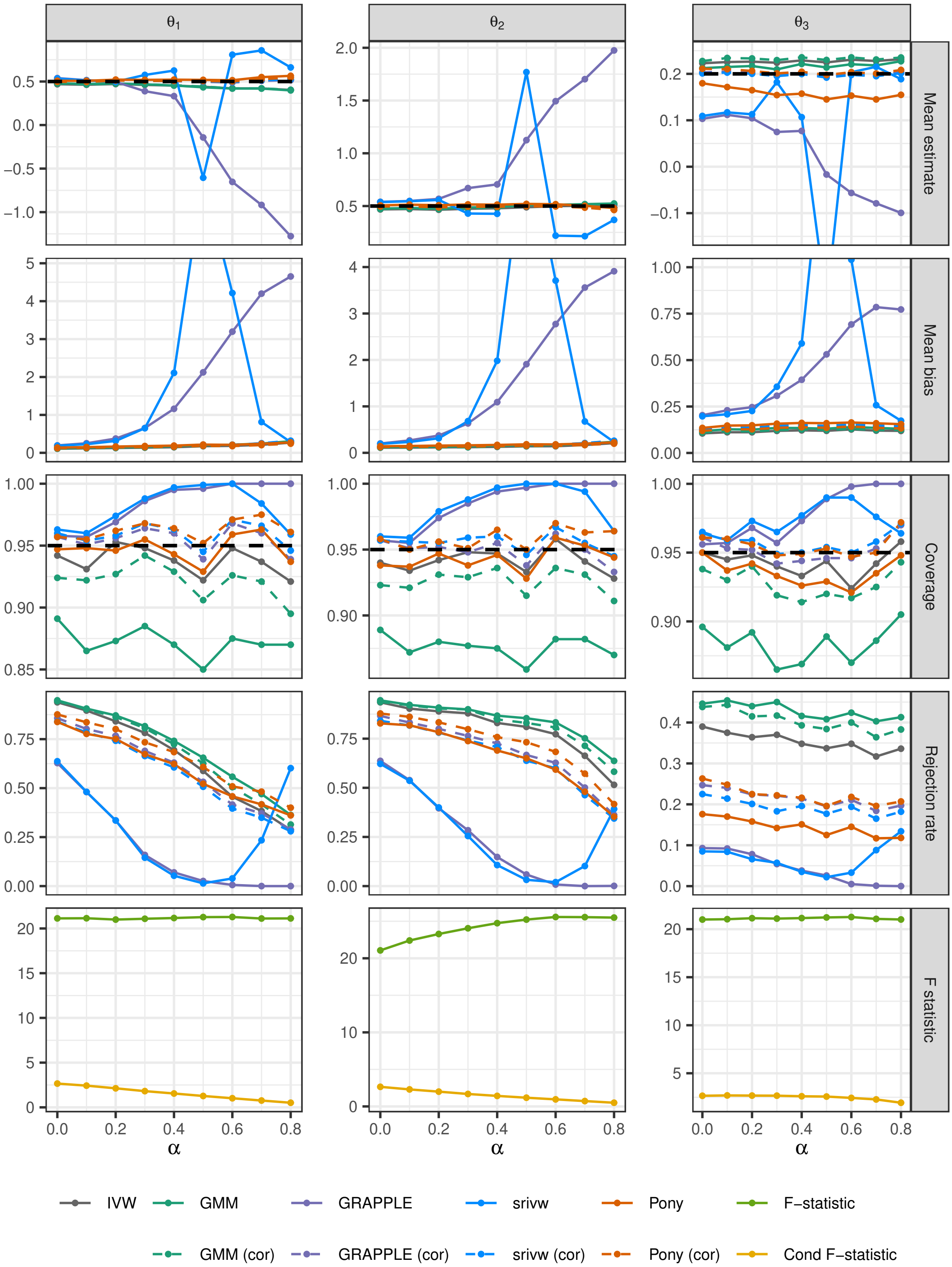

Figure S12. Simulation results for the primary simulation scenario with increasing level of mediation and increased ($K = 3$) number of exposures. Note that some values for the srivw method are beyond the displayed range.

Table S1. Simulation results for the estimation of θ_1 in the primary scenario with increasing correlated genetic effects, ρ. Shown are the mean estimates, mean bias, coverage, and rejection rate for X_1 using the IVW, GMM, GRAPPLE, srivw, and MVMR-Pony methods.

	ρ								
Method	0	0.1	0.2	0.3	0.4	0.5	0.6	0.7	0.8
	Mean estimate								
IVW	0.455	0.452	0.454	0.445	0.443	0.433	0.414	0.411	0.383
GMM	0.472	0.469	0.474	0.466	0.462	0.455	0.440	0.440	0.412
GMM (cor)	0.461	0.459	0.461	0.452	0.448	0.440	0.419	0.417	0.386
GRAPPLE	0.560	0.571	0.590	0.603	0.641	0.679	0.756	0.969	1.226
GRAPPLE (cor)	0.501	0.501	0.507	0.504	0.510	0.507	0.498	0.513	0.513
srivw	0.570	0.581	0.605	0.623	0.694	0.753	0.129	-24.257	-0.688
srivw (cor)	0.503	0.504	0.510	0.507	0.513	0.511	0.504	0.522	0.523
Pony	0.532	0.528	0.528	0.518	0.514	0.500	0.475	0.462	0.427
Pony (cor)	0.499	0.499	0.504	0.500	0.502	0.497	0.482	0.482	0.456
	Mean bias								
IVW	0.068	0.070	0.075	0.081	0.085	0.094	0.109	0.117	0.141
GMM	0.065	0.067	0.071	0.076	0.082	0.090	0.110	0.122	0.148
GMM (cor)	0.068	0.070	0.074	0.081	0.086	0.097	0.111	0.118	0.143
GRAPPLE	0.096	0.107	0.125	0.144	0.178	0.225	0.356	0.601	1.198
GRAPPLE (cor)	0.067	0.070	0.077	0.083	0.089	0.102	0.119	0.142	0.191
srivw	0.103	0.116	0.140	0.162	0.230	0.306	1.779	27.865	5.804
srivw (cor)	0.068	0.071	0.079	0.084	0.091	0.104	0.123	0.149	0.200
Pony	0.079	0.080	0.083	0.086	0.088	0.094	0.105	0.113	0.135
Pony (cor)	0.067	0.069	0.076	0.081	0.085	0.096	0.110	0.123	0.148
	Coverage								
IVW	0.903	0.906	0.908	0.894	0.897	0.898	0.860	0.875	0.854
GMM	0.908	0.915	0.909	0.895	0.891	0.887	0.840	0.831	0.806
GMM (cor)	0.901	0.909	0.903	0.885	0.875	0.875	0.836	0.844	0.829
GRAPPLE	0.959	0.964	0.944	0.959	0.967	0.979	0.989	0.998	0.999
GRAPPLE (cor)	0.965	0.956	0.950	0.951	0.953	0.955	0.950	0.976	0.964
srivw	0.952	0.959	0.946	0.955	0.960	0.978	0.995	1.000	0.995
srivw (cor)	0.968	0.956	0.954	0.954	0.955	0.959	0.955	0.977	0.974
Pony	0.948	0.955	0.935	0.937	0.944	0.956	0.944	0.960	0.938
Pony (cor)	0.961	0.954	0.944	0.943	0.951	0.954	0.945	0.969	0.953
	Rejection rate								
IVW	1.000	1.000	1.000	0.999	1.000	0.995	0.972	0.945	0.852
GMM	1.000	1.000	1.000	1.000	1.000	0.993	0.971	0.962	0.868
GMM (cor)	1.000	1.000	1.000	1.000	1.000	0.993	0.969	0.961	0.859
GRAPPLE	1.000	0.996	0.984	0.968	0.917	0.829	0.599	0.351	0.067
GRAPPLE (cor)	1.000	1.000	0.997	0.998	0.995	0.979	0.909	0.819	0.608
srivw	0.999	0.997	0.984	0.969	0.904	0.782	0.477	0.173	0.003
srivw (cor)	1.000	1.000	0.997	0.998	0.992	0.972	0.895	0.807	0.571
Pony	1.000	1.000	0.997	0.997	0.995	0.985	0.930	0.880	0.753
Pony (cor)	1.000	1.000	0.998	0.998	0.996	0.984	0.925	0.860	0.676

Table S2. Simulation results for the estimation of θ_2 in the primary scenario with increasing correlated genetic effects, ρ. Shown are the mean estimates, mean bias, coverage, and rejection rate for X_2 using the IVW, GMM, GRAPPLE, srivw, and MVMR-Pony methods.

	ρ								
Method	0	0.1	0.2	0.3	0.4	0.5	0.6	0.7	0.8
	Mean estimate								
IVW	0.025	0.029	0.030	0.036	0.037	0.046	0.067	0.072	0.102
GMM	0.012	0.016	0.013	0.019	0.022	0.028	0.046	0.047	0.077
GMM (cor)	0.027	0.030	0.030	0.037	0.039	0.046	0.070	0.073	0.106
GRAPPLE	-0.068	-0.078	-0.094	-0.110	-0.150	-0.189	-0.263	-0.474	-0.730
GRAPPLE (cor)	-0.001	-0.001	-0.005	-0.004	-0.010	-0.009	0.002	-0.011	-0.010
srivw	-0.076	-0.088	-0.109	-0.129	-0.201	-0.262	0.349	24.138	1.145
srivw (cor)	-0.003	-0.003	-0.007	-0.006	-0.013	-0.012	-0.003	-0.020	-0.018
Pony	-0.037	-0.031	-0.028	-0.020	-0.016	-0.003	0.025	0.039	0.077
Pony (cor)	0.000	0.002	-0.001	0.001	-0.003	0.001	0.019	0.020	0.048
	Mean bias								
IVW	0.059	0.061	0.069	0.073	0.077	0.084	0.099	0.109	0.132
GMM	0.060	0.062	0.071	0.074	0.079	0.085	0.104	0.118	0.144
GMM (cor)	0.062	0.065	0.073	0.077	0.082	0.091	0.105	0.115	0.139
GRAPPLE	0.098	0.110	0.129	0.146	0.184	0.231	0.361	0.605	1.198
GRAPPLE (cor)	0.065	0.068	0.079	0.083	0.090	0.100	0.119	0.141	0.193
srivw	0.105	0.119	0.142	0.163	0.234	0.312	1.792	27.288	5.801
srivw (cor)	0.065	0.069	0.080	0.084	0.092	0.102	0.123	0.148	0.201
Pony	0.078	0.080	0.085	0.086	0.089	0.093	0.106	0.113	0.137
Pony (cor)	0.064	0.067	0.077	0.081	0.087	0.094	0.111	0.124	0.149
	Coverage								
IVW	0.937	0.934	0.929	0.928	0.924	0.918	0.894	0.913	0.870
GMM	0.925	0.928	0.922	0.905	0.907	0.896	0.854	0.865	0.817
GMM (cor)	0.920	0.917	0.910	0.902	0.903	0.884	0.862	0.878	0.847
GRAPPLE	0.952	0.958	0.934	0.951	0.961	0.976	0.992	0.998	0.999
GRAPPLE (cor)	0.966	0.956	0.943	0.948	0.945	0.958	0.957	0.974	0.966
srivw	0.948	0.956	0.923	0.945	0.960	0.974	0.994	0.999	0.996
srivw (cor)	0.974	0.963	0.946	0.949	0.952	0.965	0.962	0.978	0.977
Pony	0.937	0.948	0.936	0.934	0.937	0.954	0.949	0.960	0.942
Pony (cor)	0.965	0.956	0.942	0.947	0.944	0.957	0.950	0.965	0.954
	Rejection rate								
IVW	0.063	0.066	0.071	0.072	0.076	0.082	0.106	0.087	0.130
GMM	0.075	0.072	0.078	0.095	0.093	0.104	0.146	0.135	0.183
GMM (cor)	0.080	0.083	0.090	0.098	0.097	0.116	0.138	0.122	0.153
GRAPPLE	0.048	0.042	0.066	0.049	0.039	0.024	0.008	0.002	0.001
GRAPPLE (cor)	0.034	0.044	0.057	0.052	0.055	0.042	0.043	0.026	0.034
srivw	0.052	0.044	0.077	0.055	0.040	0.026	0.006	0.001	0.004
srivw (cor)	0.026	0.037	0.054	0.051	0.048	0.035	0.038	0.022	0.023
Pony	0.063	0.052	0.064	0.066	0.063	0.046	0.051	0.040	0.058
Pony (cor)	0.035	0.044	0.058	0.053	0.056	0.043	0.050	0.035	0.046

Table S3. Simulation results for the estimation of θ_1 in the primary scenario with increasing measurement error on exposures, ν. Shown are the mean estimates, mean bias, coverage, and rejection rate for X_1 using the IVW, GMM, GRAPPLE, srivw, and MVMR-Pony methods.

	ν								
Method	0	0.5	1	1.5	2	2.5	3	3.5	4
	Mean estimate								
IVW	0.033	0.047	0.058	0.071	0.077	0.087	0.095	0.103	0.105
GMM	0.018	0.034	0.046	0.062	0.067	0.080	0.088	0.094	0.097
GMM (cor)	0.035	0.048	0.058	0.072	0.075	0.087	0.096	0.101	0.104
GRAPPLE	-0.089	-0.096	-0.109	-0.091	-0.095	-0.102	-0.099	-0.088	-0.120
GRAPPLE (cor)	-0.001	-0.003	-0.008	-0.001	-0.007	-0.008	-0.005	0.000	-0.015
srivw	-0.103	-0.113	-0.119	-0.118	-0.120	-0.103	-0.161	0.000	-0.226
srivw (cor)	-0.003	-0.005	-0.011	-0.007	-0.017	-0.019	-0.020	-0.021	-0.034
Pony	-0.025	-0.018	-0.015	-0.005	-0.002	0.006	0.010	0.022	0.017
Pony (cor)	0.002	0.002	-0.002	0.005	0.003	0.008	0.012	0.022	0.017
	Mean bias								
IVW	0.066	0.073	0.077	0.084	0.088	0.094	0.102	0.107	0.108
GMM	0.068	0.076	0.078	0.084	0.089	0.095	0.104	0.108	0.111
GMM (cor)	0.070	0.077	0.081	0.089	0.091	0.097	0.107	0.110	0.111
GRAPPLE	0.123	0.134	0.152	0.141	0.150	0.165	0.169	0.174	0.194
GRAPPLE (cor)	0.074	0.081	0.089	0.089	0.094	0.101	0.107	0.112	0.120
srivw	0.136	0.152	0.183	0.168	0.228	0.308	0.245	0.478	0.347
srivw (cor)	0.074	0.083	0.091	0.093	0.102	0.109	0.121	0.130	0.134
Pony	0.081	0.084	0.088	0.086	0.086	0.089	0.090	0.096	0.092
Pony (cor)	0.073	0.079	0.085	0.085	0.088	0.090	0.095	0.099	0.095
	Coverage								
IVW	0.927	0.895	0.862	0.833	0.814	0.761	0.716	0.642	0.668
GMM	0.919	0.875	0.851	0.814	0.782	0.729	0.668	0.626	0.618
GMM (cor)	0.912	0.869	0.839	0.797	0.777	0.724	0.642	0.615	0.610
GRAPPLE	0.958	0.968	0.972	0.983	0.990	0.994	0.994	0.989	0.990
GRAPPLE (cor)	0.964	0.959	0.963	0.956	0.961	0.961	0.963	0.969	0.957
srivw	0.954	0.964	0.972	0.977	0.985	0.995	0.994	0.991	0.995
srivw (cor)	0.972	0.961	0.964	0.955	0.959	0.968	0.968	0.974	0.965
Pony	0.958	0.949	0.958	0.949	0.954	0.950	0.958	0.951	0.959
Pony (cor)	0.962	0.957	0.959	0.948	0.944	0.955	0.952	0.954	0.951
	Rejection rate								
IVW	0.073	0.105	0.138	0.167	0.186	0.239	0.284	0.358	0.332
GMM	0.081	0.125	0.149	0.186	0.218	0.271	0.332	0.374	0.382
GMM (cor)	0.088	0.131	0.161	0.203	0.223	0.276	0.358	0.385	0.390
GRAPPLE	0.042	0.032	0.028	0.017	0.010	0.006	0.006	0.011	0.010
GRAPPLE (cor)	0.036	0.041	0.037	0.044	0.039	0.039	0.037	0.031	0.043
srivw	0.046	0.036	0.028	0.023	0.015	0.005	0.006	0.009	0.005
srivw (cor)	0.028	0.039	0.036	0.045	0.041	0.032	0.032	0.026	0.035
Pony	0.042	0.051	0.042	0.051	0.046	0.050	0.042	0.049	0.041
Pony (cor)	0.038	0.043	0.041	0.052	0.056	0.045	0.048	0.046	0.049

Table S4. Simulation results for the estimation of θ_2 in the primary scenario with increasing measurement error on exposures, ν. Shown are the mean estimates, mean bias, coverage, and rejection rate for X_2 using the IVW, GMM, GRAPPLE, srivw, and MVMR-Pony methods.

	ν								
Method	0	0.5	1	1.5	2	2.5	3	3.5	4
	Mean estimate								
IVW	0.448	0.430	0.419	0.403	0.393	0.383	0.374	0.360	0.357
GMM	0.467	0.449	0.436	0.418	0.409	0.398	0.389	0.378	0.374
GMM (cor)	0.454	0.438	0.427	0.411	0.404	0.393	0.385	0.374	0.372
GRAPPLE	0.582	0.588	0.601	0.585	0.587	0.598	0.596	0.581	0.612
GRAPPLE (cor)	0.501	0.502	0.509	0.503	0.507	0.511	0.509	0.500	0.516
srivw	0.597	0.607	0.612	0.615	0.614	0.601	0.661	0.487	0.719
srivw (cor)	0.504	0.506	0.513	0.510	0.519	0.524	0.527	0.524	0.538
Pony	0.522	0.515	0.514	0.505	0.500	0.496	0.494	0.478	0.484
Pony (cor)	0.498	0.498	0.503	0.496	0.497	0.494	0.493	0.478	0.485
	Mean bias								
IVW	0.075	0.085	0.091	0.104	0.112	0.120	0.130	0.142	0.144
GMM	0.073	0.081	0.085	0.096	0.105	0.113	0.121	0.131	0.134
GMM (cor)	0.075	0.083	0.087	0.100	0.105	0.113	0.122	0.131	0.132
GRAPPLE	0.120	0.129	0.145	0.138	0.147	0.163	0.166	0.171	0.191
GRAPPLE (cor)	0.076	0.081	0.086	0.090	0.095	0.102	0.105	0.111	0.119
srivw	0.133	0.148	0.178	0.167	0.227	0.304	0.244	0.497	0.345
srivw (cor)	0.077	0.083	0.088	0.095	0.105	0.112	0.122	0.133	0.137
Pony	0.083	0.083	0.084	0.087	0.088	0.090	0.089	0.095	0.092
Pony (cor)	0.075	0.079	0.082	0.087	0.089	0.091	0.093	0.098	0.095
	Coverage								
IVW	0.892	0.852	0.810	0.733	0.672	0.609	0.540	0.454	0.433
GMM	0.906	0.859	0.806	0.743	0.694	0.643	0.569	0.496	0.480
GMM (cor)	0.891	0.845	0.799	0.733	0.697	0.643	0.569	0.507	0.487
GRAPPLE	0.963	0.968	0.982	0.981	0.989	0.989	0.988	0.984	0.982
GRAPPLE (cor)	0.964	0.960	0.962	0.961	0.967	0.965	0.966	0.969	0.963
srivw	0.959	0.970	0.978	0.976	0.984	0.992	0.989	0.991	0.988
srivw (cor)	0.968	0.965	0.960	0.962	0.966	0.974	0.967	0.974	0.970
Pony	0.949	0.957	0.961	0.951	0.961	0.945	0.949	0.954	0.956
Pony (cor)	0.958	0.953	0.959	0.951	0.956	0.943	0.951	0.957	0.953
	Rejection rate								
IVW	1.000	1.000	0.998	0.999	0.999	0.998	0.999	1.000	1.000
GMM	1.000	1.000	0.999	0.999	0.998	1.000	0.998	1.000	0.999
GMM (cor)	1.000	1.000	0.999	0.999	0.998	0.999	0.999	1.000	0.998
GRAPPLE	0.985	0.970	0.949	0.929	0.905	0.855	0.815	0.800	0.773
GRAPPLE (cor)	1.000	0.994	0.992	0.986	0.974	0.964	0.948	0.930	0.913
srivw	0.985	0.971	0.939	0.916	0.882	0.820	0.769	0.746	0.705
srivw (cor)	0.999	0.993	0.992	0.986	0.974	0.967	0.946	0.914	0.900
Pony	0.998	0.993	0.989	0.988	0.983	0.983	0.976	0.963	0.954
Pony (cor)	0.999	0.996	0.991	0.990	0.982	0.980	0.973	0.964	0.957

Table S5. Simulation results for the estimation of θ_1 in the primary scenario with increasing level of mediation, α. Shown are the mean estimates, mean bias, coverage, and rejection rate for X_1 using the IVW, GMM, GRAPPLE, srivw, and MVMR-Pony methods.

	α								
Method	0	0.1	0.2	0.3	0.4	0.5	0.6	0.7	0.8
	Mean estimate								
IVW	0.482	0.478	0.480	0.469	0.470	0.457	0.428	0.426	0.399
GMM	0.487	0.483	0.484	0.471	0.471	0.456	0.425	0.425	0.394
GMM (cor)	0.489	0.486	0.487	0.475	0.475	0.463	0.430	0.431	0.402
GRAPPLE	0.502	0.489	0.467	0.404	0.379	0.289	-0.176	-0.435	-0.524
GRAPPLE (cor)	0.502	0.501	0.509	0.505	0.515	0.513	0.494	0.513	0.516
srivw	0.496	0.481	0.470	0.456	0.007	-0.600	1.768	0.780	0.669
srivw (cor)	0.503	0.502	0.511	0.506	0.517	0.515	0.498	0.518	0.522
Pony	0.500	0.497	0.508	0.507	0.524	0.526	0.520	0.542	0.578
Pony (cor)	0.501	0.500	0.509	0.506	0.517	0.516	0.504	0.524	0.537
	Mean bias								
IVW	0.085	0.094	0.111	0.122	0.134	0.149	0.175	0.199	0.233
GMM	0.091	0.102	0.118	0.134	0.149	0.171	0.197	0.219	0.256
GMM (cor)	0.087	0.097	0.113	0.126	0.140	0.155	0.181	0.202	0.241
GRAPPLE	0.150	0.192	0.291	0.495	0.995	1.763	2.836	3.807	4.372
GRAPPLE (cor)	0.102	0.113	0.133	0.145	0.162	0.178	0.202	0.229	0.269
srivw	0.141	0.180	0.264	0.401	1.555	4.875	4.119	1.390	0.302
srivw (cor)	0.103	0.114	0.135	0.146	0.163	0.180	0.204	0.233	0.272
Pony	0.113	0.124	0.143	0.158	0.177	0.190	0.211	0.233	0.256
Pony (cor)	0.099	0.109	0.126	0.136	0.149	0.161	0.179	0.198	0.221
	Coverage								
IVW	0.952	0.944	0.938	0.935	0.940	0.935	0.930	0.929	0.928
GMM	0.922	0.910	0.892	0.890	0.881	0.867	0.870	0.880	0.902
GMM (cor)	0.943	0.932	0.922	0.924	0.923	0.915	0.912	0.911	0.921
GRAPPLE	0.967	0.957	0.959	0.968	0.982	0.997	1.000	1.000	1.000
GRAPPLE (cor)	0.966	0.956	0.948	0.943	0.946	0.951	0.950	0.951	0.959
srivw	0.969	0.964	0.963	0.976	0.992	0.999	1.000	0.995	0.980
srivw (cor)	0.967	0.959	0.951	0.945	0.946	0.956	0.952	0.957	0.955
Pony	0.955	0.945	0.943	0.932	0.931	0.945	0.938	0.942	0.951
Pony (cor)	0.967	0.956	0.948	0.947	0.954	0.955	0.955	0.958	0.968
	Rejection rate								
IVW	0.995	0.975	0.936	0.885	0.803	0.705	0.566	0.451	0.320
GMM	0.990	0.977	0.945	0.888	0.828	0.737	0.617	0.528	0.402
GMM (cor)	0.995	0.978	0.950	0.897	0.823	0.733	0.590	0.479	0.344
GRAPPLE	0.788	0.600	0.400	0.211	0.110	0.038	0.005	0.000	0.000
GRAPPLE (cor)	0.961	0.920	0.870	0.805	0.726	0.649	0.516	0.434	0.338
srivw	0.783	0.607	0.400	0.205	0.094	0.023	0.020	0.126	0.527
srivw (cor)	0.955	0.914	0.855	0.788	0.706	0.617	0.489	0.422	0.325
Pony	0.927	0.883	0.817	0.746	0.699	0.614	0.532	0.479	0.414
Pony (cor)	0.970	0.934	0.888	0.839	0.774	0.701	0.604	0.525	0.464

Table S6. Simulation results for the estimation of θ_2 in the primary scenario with increasing level of mediation, α. Shown are the mean estimates, mean bias, coverage, and rejection rate for X_2 using the IVW, GMM, GRAPPLE, srivw, and MVMR-Pony methods.

	α								
Method	0	0.1	0.2	0.3	0.4	0.5	0.6	0.7	0.8
	Mean estimate								
IVW	0.480	0.484	0.485	0.490	0.489	0.496	0.519	0.521	0.543
GMM	0.485	0.488	0.490	0.497	0.495	0.504	0.528	0.528	0.554
GMM (cor)	0.487	0.491	0.491	0.499	0.497	0.504	0.530	0.529	0.553
GRAPPLE	0.487	0.498	0.520	0.567	0.579	0.643	0.980	1.166	1.233
GRAPPLE (cor)	0.498	0.499	0.496	0.497	0.489	0.489	0.505	0.493	0.493
srivw	0.492	0.505	0.517	0.524	0.896	1.401	-0.460	0.286	0.363
srivw (cor)	0.500	0.500	0.496	0.497	0.488	0.489	0.504	0.491	0.490
Pony	0.495	0.499	0.494	0.492	0.479	0.477	0.484	0.470	0.446
Pony (cor)	0.498	0.500	0.496	0.496	0.488	0.487	0.498	0.485	0.477
	Mean bias								
IVW	0.083	0.085	0.096	0.096	0.104	0.109	0.123	0.139	0.166
GMM	0.089	0.092	0.103	0.107	0.114	0.126	0.139	0.156	0.183
GMM (cor)	0.085	0.087	0.100	0.101	0.108	0.115	0.128	0.145	0.173
GRAPPLE	0.147	0.175	0.250	0.400	0.774	1.321	2.072	2.765	3.206
GRAPPLE (cor)	0.099	0.102	0.116	0.117	0.126	0.133	0.148	0.167	0.198
srivw	0.138	0.164	0.227	0.323	1.211	3.728	3.043	1.018	0.227
srivw (cor)	0.100	0.104	0.117	0.118	0.127	0.134	0.150	0.169	0.200
Pony	0.109	0.112	0.124	0.127	0.138	0.142	0.155	0.169	0.189
Pony (cor)	0.096	0.098	0.110	0.110	0.116	0.120	0.131	0.144	0.163
	Coverage								
IVW	0.951	0.949	0.926	0.938	0.944	0.939	0.943	0.945	0.936
GMM	0.920	0.914	0.883	0.887	0.890	0.874	0.893	0.906	0.908
GMM (cor)	0.941	0.932	0.908	0.935	0.929	0.919	0.929	0.932	0.925
GRAPPLE	0.963	0.960	0.953	0.972	0.986	0.996	1.000	1.000	1.000
GRAPPLE (cor)	0.958	0.958	0.937	0.942	0.947	0.954	0.958	0.949	0.956
srivw	0.965	0.968	0.963	0.983	0.991	0.999	1.000	0.998	0.981
srivw (cor)	0.959	0.963	0.940	0.948	0.954	0.955	0.957	0.956	0.960
Pony	0.951	0.947	0.934	0.929	0.933	0.942	0.950	0.943	0.955
Pony (cor)	0.958	0.958	0.942	0.946	0.952	0.960	0.962	0.961	0.970
	Rejection rate								
IVW	0.993	0.990	0.984	0.974	0.961	0.936	0.918	0.848	0.762
GMM	0.992	0.989	0.988	0.970	0.957	0.942	0.933	0.870	0.807
GMM (cor)	0.994	0.990	0.985	0.977	0.964	0.948	0.935	0.864	0.778
GRAPPLE	0.778	0.663	0.520	0.409	0.222	0.111	0.041	0.003	0.000
GRAPPLE (cor)	0.962	0.956	0.931	0.910	0.869	0.822	0.760	0.676	0.519
srivw	0.786	0.673	0.532	0.413	0.208	0.069	0.031	0.114	0.395
srivw (cor)	0.957	0.953	0.923	0.904	0.856	0.806	0.737	0.647	0.490
Pony	0.933	0.916	0.899	0.866	0.797	0.760	0.697	0.597	0.454
Pony (cor)	0.972	0.970	0.941	0.927	0.900	0.871	0.820	0.736	0.585
